# Alcohol Consumption and Gut Microbiota-Derived Metabolites in Primates: A Systematic Review

**DOI:** 10.3390/ijms27157012

**Published:** 2026-08-04

**Authors:** Yenny Trinidad Fierro-Salgado, Manuel Reiriz, Javier Calleja-Conde, Clara Cintado-Alzate, Kora-Mareen Bühler, José A. Morales-García, Jose A. López-Moreno, Elena Giné, Víctor Echeverry-Alzate

**Affiliations:** 1 Basic and Clinical Neuroscience Research Group (NBC), School of Life and Nature Sciences, Nebrija University, 28240 Madrid, Spain; yfierro@nebrija.es (Y.T.F.-S.); mreiriz@nebrija.es (M.R.); jcalleja@nebrija.es (J.C.-C.); ccintadoa@alumnos.nebrija.es (C.C.-A.); 2 Department of Psychobiology and Methodology in Behavioral Sciences, Faculty of Psychology, Complutense University of Madrid, 28223 Madrid, Spain; kobuhler@ucm.es (K.-M.B.); jalopezm@psi.ucm.es (J.A.L.-M.); 3 Biomedical Research Networking Center for Frailty and Healthy Aging (CIBERFES), Carlos III Health Institute, 28031 Madrid, Spain; jamorales@ucm.es; 4 Department of Cell Biology and Histology, Faculty of Medicine, Complutense University of Madrid, 28040 Madrid, Spain; elena.gine@med.ucm.es

**Keywords:** alcohol use disorder, gut microbiome, microbiota-derived metabolites, metabolomics, short-chain fatty acids, tryptophan metabolism, gut–brain axis

## Abstract

Alcohol consumption has been increasingly associated with alterations in the gut microbiota and its metabolic activity; however, evidence regarding microbiota-derived metabolites remains fragmented. This systematic review aimed to synthesize current evidence on the effects of alcohol consumption on gut microbiota-derived metabolites in humans and non-human primates. The review was conducted according to Preferred Reporting Items for Systematic Reviews and Meta-Analyses (PRISMA) guidelines and included studies published between 2012 and 2026. Searches were performed in PubMed, Web of Science, Scopus, and ScienceDirect. Study quality was assessed using the Newcastle–Ottawa Scale for human studies and the Systematic Review Centre for Laboratory Animal Experimentation (SYRCLE) Risk of Bias tool for non-human primate studies. Twelve studies met the inclusion criteria, comprising four non-human primate studies and eight human studies. Alcohol exposure was consistently associated with metabolomic alterations across multiple biological matrices. Recurrent findings included reductions in short-chain fatty acids, alterations in tryptophan-derived metabolites, changes in phenolic and aromatic amino acid-related compounds such as hippuric acid, and disturbances in bile acid and purine metabolism. Findings regarding microbial diversity and taxonomic composition were more heterogeneous, with several studies reporting reduced abundances of *Faecalibacterium* and related butyrate-producing taxa. Studies evaluating abstinence suggested partial recovery of both microbial and metabolomic alterations. Overall, the available evidence suggests that alcohol consumption is associated with alterations across several microbiota-related metabolic pathways, highlighting candidate metabolites that may contribute to alcohol-related pathophysiology and serve as potential translational biomarkers.

## 1. Introduction

Alcohol consumption is a practice strongly embedded in many cultures, with more than 2.3 billion consumers around the world [1], and excessive alcohol consumption has been associated with health problems worldwide [2]. In this sense, according to the World Health Organization, alcohol abuse accounted for 4.7% of all deaths in 2024 [3]. Regarding drinking patterns, two main types of excessive alcohol consumption can be identified, each with distinct health consequences. Firstly, binge drinking is characterized by the intake of five drinks for males or four for females within about two hours [4]. Secondly, a more chronic pattern, such as heavy weekly drinking, is defined as the consumption of five or more drinks per day or 15 per week, whereas the corresponding thresholds for females are four drinks per day and eight per week, respectively [5].

Traditionally, alcohol abuse has been associated with damage to the brain and liver; thus, alcohol consumption is one of the main causes of liver disease worldwide and can lead to alcohol-related liver disease, characterized by fatty liver, alcoholic hepatitis, steatohepatitis, and progression to fibrosis and cirrhosis [6,7]. Moreover, alcohol consumption can produce other types of damage, including neuroinflammation, metabolic alterations, cardiovascular dysfunction, and immune dysregulation, as well as behavioural and psychiatric alterations [8,9,10].

All these negative effects are mediated by multiple underlying mechanisms, such as the disruption of metabolic pathways across multiple organs by alcohol metabolites, inducing oxidative stress and depleting nicotinamide adenine dinucleotide, thereby impairing energy-generating pathways [11], structural damage to mitochondria or microtubules caused by acetaldehyde (an alcohol metabolite), and the overproduction of reactive oxygen species (ROS), which promotes oxidative stress and inflammation [12,13,14]. Finally, excessive alcohol consumption is associated with different comorbidities such as type 2 diabetes mellitus, cardiovascular disease, obesity and malnutrition, and neurological damage, as well as alcoholic myopathy and muscle wasting [15].

In addition to these well-established mechanisms, increasing evidence suggests that alcohol consumption also disrupts gut microbiota homeostasis, emerging as a key contributor to alcohol-induced systemic dysfunction [16,17].

The human microbiome comprises a complex community of microorganisms, including bacteria, viruses, fungi and yeasts, which inhabit different sites of the body. Among them, the gut microbiota represents the largest and most diverse microbial community, accounting for the majority of microorganisms present in the human organism [18,19,20]. These microorganisms play a critical role in a wide array of physiological processes, including nutrient absorption and the maintenance of the intestinal mucus layer, which serves as a protective barrier [21,22].

In addition, the gut microbiota has the capacity to modulate central nervous system (CNS) function through a bidirectional pathway that connects the gastrointestinal tract and the CNS, known as the microbiota–gut–brain axis [18]. Thus, for example, the gut microbiota can influence CNS activity through the stimulation of the vagus nerve [23], the modulation of the activation of the hypothalamic–pituitary–adrenal axis [24], or the production of metabolites that can cross the blood–brain barrier (BBB) and directly affect CNS activity [25].

In this sense, the gut microbiota composition has a significant impact on CNS functioning. Healthy microbiota is characterized by high richness and diversity in its composition, with Bacteroidetes and Firmicutes as the predominant phyla, followed by Actinobacteria and Verrucomicrobia [26]. However, the disruption of the gut microbiota, known as dysbiosis, which is often characterized by an overgrowth of Gram-negative bacteria [27], is associated with various pathologies such as mental disorders, obesity, gastrointestinal disorders, CNS disorders, and even neurodegenerative diseases, as well as conditions like irritable bowel syndrome and immune-related diseases, including allergies, autoimmune diseases, and inflammatory bowel disease [28,29,30,31,32].

Importantly, the gut microbiota is a highly dynamic ecosystem that can be modulated by multiple lifestyle and environmental factors throughout life, including antibiotic use, physical activity, sleep patterns, circadian rhythms, and diet [33]. Moreover, alcohol is included in the diet of millions of people around the world and is, together with caffeine, one of the most widely consumed psychoactive substances in our societies [34].

Alcohol toxicity can directly induce dysbiosis in the gut microbiota through different mechanisms, such as increased fecal pH, alterations in gastric acid secretion, bile acid metabolism, and gut motility [35,36,37,38]. Indeed, heavy and chronic alcohol consumption has been shown to reduce the biodiversity of gut microbes, particularly affecting specific taxa, including a reduction in the Bacteroidetes and Firmicutes phyla and an increase in Proteobacteria, thereby promoting intestinal inflammation [39,40,41]. On the other hand, binge drinking patterns have not shown homogeneous or consistent results [42,43].

In addition, alcohol abuse disrupts intestinal barrier integrity and damages the intestinal mucosa, as well as reduces the expression of tight junction proteins, increasing gut permeability [44,45,46,47,48,49]. This increased permeability may facilitate the translocation of bacteria or critical microbial components into the bloodstream [39]. One of these components is lipopolysaccharide (LPS), a Gram-negative bacterial membrane product that acts as an endotoxin and can increase the production of pro-inflammatory cytokines, leading to chronic inflammation not only in the gut but also at the systemic level, including the brain [39].

Moreover, alcohol abuse alters the production of neuroactive compounds by the gut microbiota, which can influence host behaviour [50]. Thus, alcohol reduces the production of short-chain fatty acids (SCFAs), such as butyrate, propionate and acetate, by decreasing the abundance of microbial taxa responsible for their synthesis. SCFAs participate in the maintenance of epithelial and BBB integrity, promote the production of anti-inflammatory cytokines, modulate the immune system and regulate the gut–brain axis. The reduction in SCFA production leads to impaired epithelial integrity and contributes to systemic inflammation [51,52,53]. 

Other metabolites altered by alcohol abuse are gut microbiota-derived tryptophan compounds (such as indole, indolic acid, skatole, tryptamine, and kynurenine). These metabolites are critical for gut homeostasis and systemic immunity [54]. Thus, tryptophan derivatives participate in neuroimmune regulation by promoting the function of interleukin (IL)-22, anti-inflammatory macrophages, regulatory T cells, CD4+CD8αα+ regulatory cells, and IL-10+ and/or IL-35+ B regulatory cells [54]. Tryptophan metabolites also contribute to the maintenance of intestinal homeostasis by acting as ligands of the aryl hydrocarbon receptor (AhR), thereby promoting immune homeostasis [55].

Finally, alcohol abuse affects the production of bile acids. These acids play a key role in intestinal barrier function through two main bile acid receptors, farnesoid X receptor (FXR) and G protein-coupled bile acid receptor 1 (TGR5). Bile acids are crucial for lipid absorption and are biotransformed by the gut microbiota [56]. Moreover, bile acids act as systemic endocrine signals and regulate the pro-inflammatory/anti-inflammatory balance [57], and their alteration has been associated with diseases such as cardiometabolic disorders, inflammatory diseases, liver disease, and neoplastic conditions [58,59]. Alcohol consumption alters bile acid metabolism, composition, and signaling, contributing to gut–liver axis dysfunction [60]. Thus, alcohol consumption disrupts bile acid-mediated signaling pathways, including FXR and TGR5, which play key roles in metabolic regulation, inflammation and gut–liver communication [61].

In addition, alcohol consumption has been associated with alterations in bile acid pool size and composition, including increased systemic bile acid levels and disrupted enterohepatic homeostasis [62]. Furthermore, alcohol-related liver disease disrupts bile acid synthesis, transport and enterohepatic circulation, leading to altered bile acid profiles in both plasma and intestinal compartments [63]. Finally, alcohol-induced dysbiosis alters microbial bile acid transformation, leading to changes in the production of secondary bile acids and contributing to gut–liver axis dysfunction. Thus, disruption of bile acid homeostasis contributes to inflammation, metabolic dysfunction and the progression of alcohol-related liver disease [64].

Although most evidence is derived from rodent models, an increasing number of studies in non-human primates have begun to explore alcohol-induced alterations in gut microbiota and associated metabolic pathways, highlighting their potential translational relevance [65].

Although several reviews have summarized the effects of alcohol consumption on gut microbiota composition and its role in alcohol-related diseases, considerably less attention has been paid to gut microbiota-derived metabolites, which represent the functional output of microbial activity and may provide more direct insights into the biological mechanisms linking alcohol exposure to host physiology. Furthermore, evidence from non-human primates has not been systematically integrated with human studies, despite its particular translational relevance.

Despite these advances, the available evidence remains fragmented. Numerous studies have investigated the impact of alcohol consumption on gut microbiota composition, liver disease, neuroinflammation, and metabolomic alterations; however, no previous systematic review has specifically synthesized the evidence on gut microbiota-derived metabolites across both humans and non-human primates. Although humans and non-human primates exhibit species-specific differences in gut microbiota composition, alcohol metabolism, and behavioural responses, they share many fundamental microbiota-associated metabolic functions, supporting the translational relevance of functional microbiome comparisons across primates [66,67]. Consequently, focusing on microbiota-derived metabolites may provide a more robust and functionally relevant basis for cross-species comparisons than taxonomic composition alone, offering physiologically relevant insights into alcohol-related pathophysiology.

This systematic review aims to synthesize current evidence on the effects of alcohol consumption on gut microbiota-derived metabolites in humans and non-human primates. Special attention is given to metabolomic alterations associated with alcohol exposure, their relationship with changes in gut microbiota composition when assessed, and their evolution during abstinence. By integrating findings from both human and non-human primate studies, this review seeks to identify recurrent microbiota-related metabolic signatures associated with alcohol consumption and to provide a translational overview of the potential links between alcohol exposure, gut microbial metabolism, and alcohol-related pathophysiology.

## 2. Materials and Methods

### 2.1. Protocol and Registration

The systematic review protocol was registered with the Open Science Framework (OSF) before completion of the data synthesis and analysis procedures. The registration DOI was: https://doi.org/10.17605/OSF.IO/B82M4 (accessed on 23 May 2026).

### 2.2. Literature Search

The present systematic review was conducted in accordance with the Preferred Reporting Items for Systematic Reviews and Meta-Analyses (PRISMA) guidelines [68,69]. A systematic literature search was performed to identify studies published between 2012 and 2026. This time range was established following preliminary exploratory searches across multiple databases, which indicated that the relevant literature on alcohol-associated gut microbiota-derived metabolites became more consistently available during this period. Searches were carried out using Web of Science, ScienceDirect, PubMed, and Scopus, selected for their broad coverage of the biomedical, microbiome, metabolomics, and translational research literature relevant to the objectives of this review. Two authors (Y.T.F.-S. and V.E.-A.) independently conducted the literature search between January and February 2026 and updated it between April and May 2026. They also independently performed the initial screening of titles and abstracts, followed by a full-text assessment to determine study eligibility. The search strategy was adapted for each database and included the following terms: “alcohol consumption” AND (“gut” OR “gut microbiome” OR “gut microbiota”) AND (“metabolome” OR “metabolomics”). Additionally, relevant studies identified through citation tracking during full-text evaluation were assessed for eligibility.

### 2.3. Study Selection

The studies analyzed in this systematic review were screened for inclusion according to the following eligibility criteria: (i) involvement of humans or non-human primates; (ii) examination of alcohol consumption or experimental alcohol exposure; (iii) assessment of alcohol-related gut microbiota-derived metabolites; (iv) evaluation of gut microbiota composition, when reported; (v) availability of the full text online; and (vi) publication as original research articles in English between 2012 and 2026.

The exclusion criteria included the following: (i) reviews, meta-analyses, editorials, commentaries, conference abstracts, protocols, or letters; (ii) studies conducted exclusively in non-primate animal models; (iii) studies focused exclusively on severe alcohol-related comorbidities or alcohol-related liver disease when metabolomic alterations could not be differentiated from disease-specific pathological processes underlying these conditions; (iv) articles for which the full text was not available; and (v) insufficient information regarding alcohol exposure or metabolomic assessment.

**Figure 1:**
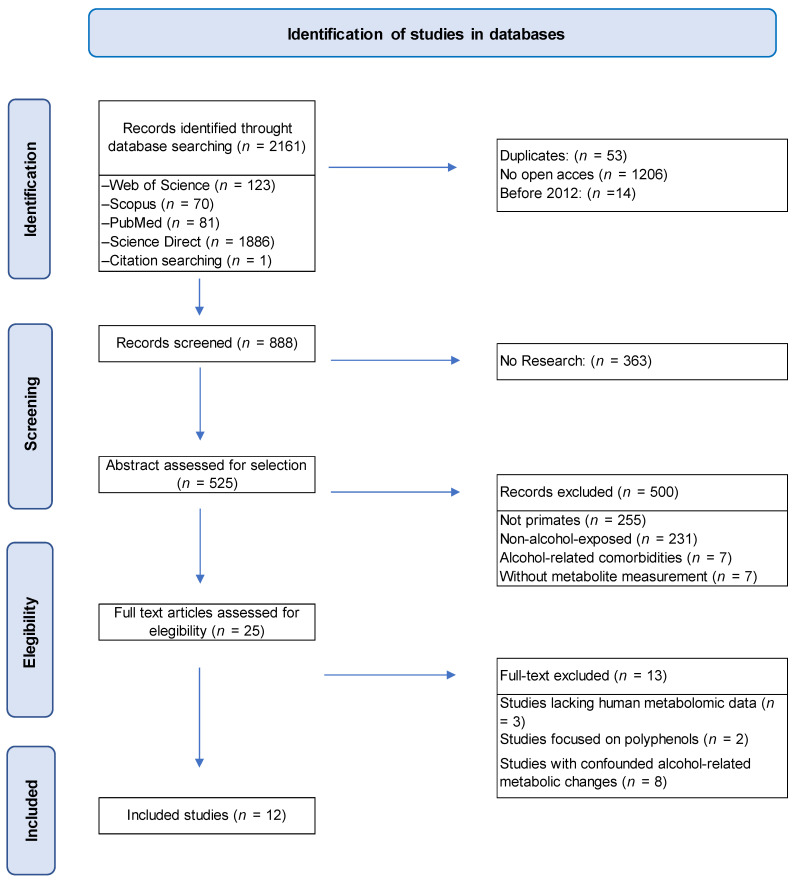
Flow diagram of the search and selection process. A total of 12 studies met the eligibility criteria and investigated the effects of alcohol consumption on gut microbiota-derived metabolites in humans (*n* = 8) and non-human primates (*n* = 4).

Initially, a total of 2161 records were retrieved (Figure 1). Duplicate records, non-open-access articles, and records published before 2012 were excluded. Next, 888 articles were selected based on their titles. Of these, 363 studies were excluded because they were not original research (e.g., reviews, editorials, commentaries, conference abstracts, protocols, and letters), leaving 525 records for abstract screening. During the abstract screening, 500 records were excluded because they did not include primates, did not involve alcohol exposure, involved severe alcohol-related comorbidities in which metabolomic alterations could not be distinguished, or lacked metabolite measurements. The remaining 25 full-text articles were assessed for eligibility. Of these, 13 were excluded: (i) studies with metabolomic data conducted solely in non-primate models (*n* = 3), (ii) studies focusing on polyphenols rather than ethanol (*n* = 2), and (iii) studies with alcohol-related metabolic changes that introduced confounding factors (*n* = 8). Finally, 12 studies met the inclusion criteria and were included in the final synthesis. 

## 3. Results

### 3.1. Data Extraction

After selecting the articles, the included studies were reviewed to extract data for narrative synthesis (Table 1). Data extraction focused on the following areas:

- Study population and species (humans or non-human primates), sample size, sex, and age.
- Comparison of groups (e.g., alcohol-exposed vs. control, consumption vs. abstinence, stratification by level of consumption).
- Characteristics of alcohol exposure: pattern, duration, dose/volume, and measurement method.
- Type of biological sample and analytical platforms used for microbiota profiling and metabolomic profiling.
- Microbiota-related results: described taxa and changes in composition.
- Metabolomic results: altered metabolites and affected metabolic pathways.
- Functional consequences: physiological and behavioral outcomes associated with alcohol-related alterations.

A total of 12 studies met the inclusion criteria and were included in the qualitative synthesis. Of these, four were conducted in non-human primates [65,70,71,72] and eight in human populations [39,73,74,75,76,77,78,79]. The studies were published between 2014 and 2026.

Regarding population characteristics, human studies included participants with alcohol dependence (AD) [39,74], severe alcohol use disorder (AUD) [79], individuals with AUD including both actively drinking and abstinent participants [78], and chronic heavy drinkers [75,76,77], as well as cohorts from the general population and individuals with major depressive disorder stratified by alcohol consumption [73]. Sample sizes ranged from 31 to 502 participants. The studies involving non-human primates had sample sizes ranging from 14 to 63 animals.

Alcohol exposure was assessed via self-report in all human studies, using the Timeline Follow-Back [77,78], the Alcohol Use Disorders Identification Test [74], Diagnostic and Statistical Manual of Mental Disorders (DSM)--based criteria [39,74], or quantity–frequency questionnaires [73,75,77]. In non-human primate studies, alcohol exposure was experimentally controlled using operant self-administration paradigms with daily access to ethanol solutions and blood alcohol concentration monitoring. Fecal samples were the primary biological matrix (*n* = 11), with additional plasma [71,74,79], serum [73], and post-mortem frontal cortex and cerebrospinal fluid samples [79]. Microbiota was assessed by 16S rRNA sequencing (*n* = 7) or shotgun metagenomic sequencing [75]. Metabolomics employed untargeted liquid chromatography–tandem mass spectrometry (LC-MS/MS) (*n* = 7), targeted LC-MS/MS (*n* = 1), and gas chromatography–mass spectrometry (GC-MS) (*n* = 6).

Potential confounding variables were addressed differently across the included studies. In the four studies involving non-human primates [65,70,71,72], these factors were largely minimized through standardized housing conditions, controlled diets, and longitudinal designs in which animals served as their own controls. In addition, sex was explicitly considered in two studies [71,72] and age in one [70]. In human studies, basic demographic characteristics, including age and sex, were reported in most studies [39,73,75,76,78,79]. However, the assessment and control of other potential confounding variables were inconsistent. Nutritional status was evaluated in one study using the Mini Nutritional Assessment [76]. Medication use and medical comorbidities were explicitly considered in one study [78]. Smoking status was adjusted for in one study [73], acknowledged as a potential uncontrolled confounder in another [79], and not reported in the remaining studies [39,75,76,77]. Likewise, psychiatric comorbidities, particularly depression and anxiety, were primarily evaluated as clinical outcomes rather than controlled as potential confounding factors in several studies [39,73,74,78,79]. Finally, one study did not report any adjustment for potential confounding variables [77].

**Table 1:** Effects of alcohol consumption on gut microbiota composition, microbiota-derived metabolites, and functional outcomes in primates.

Model/Population	Groups Compared and Alcohol Exposure	Alcohol Measurement Method	Biological Sample/Method	Microbiota Results	Metabolomic Results	Functional Consequences	No.
Non-human primates (NHPs)							
*Macaca mulatta*, *n* = 63 (baseline vs. 12 months alcohol), adult.	Longitudinal: baseline vs. 12 months alcohol Exposure: Chronic; 12 months daily voluntary access (22 h/day); 4% *w*/*v* ethanol	Operant panel (daily volume/dose) + Blood Ethanol Concentration (BEC).	FM: 16S rRNA (V4). Met: feces and plasma; untargeted LC-MS + targeted GC-MS for SCFAs and fatty acids.	↓ SCFA producers: Prevotellaceae, Muribaculaceae, Rikenellaceae, Christensenellaceae, Bacteroidales, Clostridia. ↑ carbohydrate-fermenting bacteria: *Colidextribacter*, *Succinivibrio*, *Eubacterium*, *Monoglobus*. ↓ Firmicutes and Bacteroidetes. *Subdoligranulum* positively correlated with BEC; Bradymonadales negatively correlated with BEC.	Feces: ↑ LCA, proline, quinolinate, G1P, TMP, XMP, UMP, CMP. Feces (SCFA): ↓ acetate, propionate, isobutyrate, n-butyrate, 2-methylbutyrate, isovalerate, n-valerate. ↓medium/long-chain FA (palmitic, oleic, stearic, etc.). Plasma: ↑ glycochenodeoxycholate, isoleucine, phenylalanine, proline, tyrosine, arginine; ↓pipecolate, cortisol, corticosterone. Enrichment: ↑ fatty acid oxidation; ↓ purine/pyrimidine metabolism, steroidogenesis.	Immunological: fecal metabolites from 12-month drinking animals enhanced monocyte inflammatory response to LPS (↑ TNFα and IL-6 production) vs. controls, indicating potential “innate immune training”.	[72]
*Macaca mulatta*, *n* = 31–40 (alcohol group: baseline vs. 6 months; maltose controls for temporal changes), adult.	Longitudinal: ethanol-naïve baseline vs. 6 months alcohol (same subjects); maltose controls for temporal changes. Exposure: Chronic; 6 months daily voluntary access (22 h/day, 7 days/wk); 4% *w*/*v* ethanol; maltose self-administration controls.	Operant panel (daily volume/dose); BAL not reported.	FM: 16S rRNA(V4). Met: feces and plasma; untargeted HILIC LC-MS + targeted GC-MS for SCFAs and fatty acids.	↓ SCFA producers: *Eubacterium*, *Subdoligranulum*, *Lactobacillus*, Butyricoccaceae; ↑ *Prevotella*, *Faecalibacterium*, *Dorea*, *Sutterella*. Dose-dependent: ↑ *Lactobacillus*, *Terrisporobacter*, *Colidextribacter* with higher intake; ↓ *Clostridium*, *Catenibacterium*, *Sarcina*, Oscillospiraceae.	Feces: ↑ DCA, mercaptopyruvic acid, sulfuric acid, urocanic acid; ↓ tyrosine, N-methylproline, Gly-Pro, propionic acid (SCFA), and medium/long-chain FA (dodecanoic, palmitic, oleic, stearic, etc.). Plasma: ↑ acetylaurine, β-hydroxybutyric acid, stachyridine, urate, kynurenate, guanine, guanosine, glutamic acid. Enrichment: ↑ fatty acid oxidation, purine metabolism, tryptophan metabolism.	N/A	[71]
*Macaca mulatta*, *n* = 21 (Adolescents alc = 6/CT = 6; Adults alc = 4/CT = 5), 4–6 y/10–15 y.	Longitudinal: baseline vs. 5-day abstinence within alcohol group + cross-sectional: alcohol vs. CT. Exposure: Chronic; adolescents: 0.5–1.5 g/kg/day, 3 months; adults: 0.8–2.2 g/kg/day, 5 years; 4% *w*/*v* ethanol; 5 days/week.	Operant panel (daily volume/dose) + BAL (AnaloxGM7).	FM: 16S rRNA (V4). Met: feces; untargeted UPLC-MS/MS and GC-MS.	↓ alpha-diversity in alcohol vs. CT; ↑ Firmicutes (Streptococcaceae) and ↓ Bacteroidetes in drinking; partial recovery after abstinence. Alcohol-ever taxa reduced: Euryarchaeota, Tenericutes, Lentisphaerae, Verrucomicrobia.	22 metabolites changed (drinking vs. abstinence): ↑ fatty acids (myristoleate, linolenate, arachidonate) in drinking; ↓ glycolysis intermediates (glucose-6-phosphate, fructose-6-phosphate) in drinking; no significant differences between control and drinking.	N/A	[70]
*Papio anubis*, *n* = 14. (L, *n* = 4; S, *n* = 5; CT, *n* = 5), L 17.5 (IQR 1.7) y/S 15.0 (IQR 3.5) y/C 15.0 (IQR 2.5) y	Cross-sectional: L vs. S vs. C; longitudinal: Drinking (D, 3 days) vs. Abstinence (A, first 3 days of withdrawal) within groups. Exposure: Chronic; L: 12.1 years, S: 2.7 years, C: Tang control; 0.63–1.15 g/kg/day, 7 days/wk.	Operant self-administration (CSR) + BAL (AnaloxGM7).	FM: 16S rRNA (V3–V4). Met: feces; untargeted UPLC-MS/MS.	L vs. S and C: ↓ alpha-diversity, ↓ beta-diversity, ↓ *Prevotella*, *Blautia*, *Dialister*, *Sutterella*; ↑ *Lactobacillus*, *Streptococcus*. L vs. C: ↓ *Faecalibacterium*, *Parabacteroides*, *Oribacterium*, *Collinsella*, *Desulfovibrio*; ↑ *Lactobacillus*, *Streptococcus*. S vs. C: minimal differences. Condition D vs. A: no major changes.	L vs. S and C: ↑ aromatic amino acid metabolites (N-acetylphenylalanine, phenylacetate, indoleacetate), ↑ methionine cycle metabolites, ↑ 2-hydroxybutyrate, ↑ pentose metabolites, ↑ TCA metabolite alpha-ketoglutarate; ↑ N-acetylated amino acids. Condition D vs. A: metabolome shifted between conditions.	N/A	[65]
Humans—Microbiota and Metabolomics							
Human, *n* = 75. AD patients *n* = 60 (subset *n* = 13 for microbiota/metabolome) and HC *n* = 15; no significant fibrosis/cirrhosis; 48 ± 11 y/48 ± 10 y	Cross-sectional: AD high Intestinal Permeability (IP) vs. AD low IP vs. HC; longitudinal: T1 (admission) vs. T2 (day19 abstinence) within AD. Exposure: Chronic heavy; AD: mean 188 g/day (high IP)/177 g/day (low IP); HC: 11 ± 7 g/day.	Self-report: TLFB.	FM: 16S rRNA (V1–V2) pyrosequencing + qPCR. Met: feces; VOCs by GC-MS.	AD high IP vs. low IP/CT: ↓ total bacteria, ↓ Ruminococcaceae (*Ruminococcus*, *Faecalibacterium*, *Subdoligranulum, Oscillibacter*, *Anaerofilum*), ↓ Incertae SedisXIII, ↑ Lachnospiraceae (*Dorea*), ↑ Incertae Sedis XIV, ↑ *Blautia*, ↑ *Megasphaera*, ↓ *Bifidobacterium*. IP negatively correlated with total bacteria, *F. prausnitzii*, and *Bifidobacterium*; positively with *Dorea* and *Blautia*. Partial recovery after 3-week abstinence: ↑ total bacteria, *Bifidobacterium*, *Lactobacillus*; *F. prausnitzii* remained low.	High IP vs. low IP/controls: ↑ phenol; ↓ 4-methylphenol (p-cresol), ↓ indole, ↓ 3-methylindole in high IP. 2-methyl-1-butanol and methanethiol present in controls, absent in AD. Alcohols, alkanes, benzenes present only in AD.	Immunological: high IP had higher plasma IL-8, negatively correlated with *F. prausnitzii.* Physiological: IP recovered after 3-week abstinence, but *F. prausnitzii* remained low. Behavioral: high IP had higher depression, anxiety, and craving at T2 vs. low IP/controls. IP at T1 positively correlated with depression, anxiety, and craving at T2. Low IP group recovered completely in depression and anxiety at T2.	[39]
Human, *n* = 502 surgical patients (CT *n* = 354; Low-frequency drinking (L) *n* = 117; High-frequency drinking (H) *n* = 31), 48.8 ± 15.8 y	Cross-sectional: control vs. L vs. H. Exposure: Chronic; L: ≥2 drinks/wk; H: daily ≥23 g ethanol; Control: Non-drinkers: ≤2 drinks/year.	Self-report(frequency categories).	FM: shotgun metagenomic sequencing. met: feces; untargeted UHPLC-Q-TOF MS.	Human alcohol vs. control: ↓ *F. prausnitzii* C/G, ↑ *F. prausnitzii* E, ↑ *Enterocloster bolteae.*	Alcohol-exposed (L + H) vs. control: ↑fecal adenosine.	Physiological: prolonged alcohol exposure reduces anesthetic efficacy (prolongs induction time and increases anesthetic dose required) in surgical patients.	[75]
Human, *n* = 42 (alcohol overconsumers *n* = 24; CT *n* = 18), 64.8 (43–85) y/58.2 (34–78) y.	Cross-sectional: alcohol overconsumers vs. CT. Exposure: Chronic heavy; ≥20 g/day (women)/≥40 g/day (men), >10 years, mean 118.9 g/day; CT: ≤5 g/day(women)/≤10 g/day(men).	Self-report (TLFB).	FM: 16S rRNA(V3–V4). Met: feces; SCFAs by vacuum distillation + GC-FID.	↑ Proteobacteria (Enterobacteriaceae, Desulfovibrionaceae), ↑ *Sutterella*, ↑ *Holdemania*, ↑ *Clostridium*; ↓ *Faecalibacterium*. No alpha-diversity difference; beta-diversity difference. Proteobacteria inversely correlated with butyric acid; *Faecalibacterium* positively correlated with butyric acid.	Alcohol overconsumers vs. controls: ↓ butyric acid percentage and concentration. No significant differences in other SCFAs. Proteobacteria inversely correlated with butyric acid, acetic acid, valeric acid, and total SCFAs. *Faecalibacterium* positively correlated with butyric acid.	Physiological: lower handgrip strength, lower Mini Nutritional Assessment (MNA) scores, decreased plasma vitamin C levels in alcohol overconsumers.	[76]
Human, *n* = 31. AB (AUD) *n* = 10; CD (AUD) *n* = 9; HC *n* = 12. 45.9 ± 11.4 y/45.0 ± 12.6 y/48.8 ± 12.0 y	Cross-sectional: AB vs. CD vs. HC. Exposure: Chronic heavy; AB: prior 13.6 drinks/day, abstinent ≥6 wks; CD: 3.6 drinks/day during study; HC: ≤1–2 drinks/day.	Self-report (TLFB, AUDIT, ADS).	FM: 16S rRNA (V3–V4). Met: feces; untargeted LC-MS.	AB vs. CD and AB vs. HC: ↓ *Akkermansia*, *Lachnospira*, *Roseburia*, *Fusicatenibacter*, *Lachnospiraceae_UCG_001*; ↑ *Streptococcus*, *Eisenbergiella*, *candidatus Stoquefichus*. No consistent differences between CD and HC.	AB vs. CD and AB vs. HC: ↓ tryptophan metabolites (skatol, xanthurenate), ↓ oxalate, ↓ L-urobilin; ↑ lipids (hexosylceramides, lactosylceramides, ceramides), ↑ fructosyllysine, cystine, carboxymethylarginine, 1-methylxanthine. CD vs. HC: minimal differences.	Physiological: AB individuals had greater sleep disturbance, more medical comorbidities, and more medications compared to CD and HC. Behavioral: AB individuals showed higher depression and higher anxiety.	[78]
Humans—Metabolomics-only							
Human, *n* = 81 (endoscopy collected: alcoholics *n* = 16, HC *n* = 18; home collected: alcoholics *n* = 22, HC *n* = 25), 20–64 y.	Cross-sectional: alcoholics vs. HC. Exposure: Chronic heavy; alcoholics: women ≥ 4 drinks/day or ≥8 drinks/wk, men ≥ 5 drinks/day or ≥15 drinks/wk; controls: women < 2 drinks/sitting or ≤7 drinks/wk, men < 4 drinks/sitting or ≤14 drinks/wk.	Self-report questionnaire.	FM: not assessed. Met: feces; VOCs by hSPME + GC-MS.	N/A	Endoscopy-collected (alcoholics vs. controls): ↑ tetradecane; ↓ fattyalcohols (2-tetradecen-1-ol, 1-undecanol, 6-pentadecen-1-ol, 1,15-pentadecanediol, eicosen-1-ol), ↓propanoic acid, ↓ cyclopropanenonyl-, ↓ 8-tetradecen-1-yl acetate. Home-collected: ↓ caryophyllene, ↓ 1-naphthalenol, ↓ phellandrene, ↓ dimethyl disulfide, ↓ dimethyltrisulfide, ↓ camphene, ↓ 2,5-pyrrolidinodiene, ↓ 5-hepten-2-one, ↓ (2-aziridinylethyl) amine.	Physiological (inferred): elevated fecal tetradecane suggested increased intestinal oxidative stress; decreased fatty alcohols may reflect reduced plasmalogen synthesis; decreased propionate may compromise intestinal barrier integrity.	[77]
Human, *n* = 346 P) *n* = 247 and MDD *n* = 99), 54.6 ± 9.5 y/39.4 ± 11.9 y.	Cross-sectional: alcohol intake stratified (light vs. moderate vs. heavy) within GP and MDD cohorts. Exposure: Habitual; light (<6 drinks/wk), moderate (6–21), heavy (>21); 1 drink = 12g ethanol; mean intake among consumers: 10.9–10.2 g/day.	Self-report (questionnaire, 8 categories).	FM: not assessed. Met: serum; targeted LC-MS/MS.	N/A	Significant correlations with alcohol use: GP cohort: ↓ hippuric acid, ↑ chenodeoxycholic acid; MDD cohort: ↓ xanthosine. Trends: GP: ↓ glycine, serine, glutamine, 5-HIAA, ↑ cytidine, taurocholic acid; MDD: ↑ GABA, cytidine, 2-aminoisobutyric acid, ↓ carnosine.	Behavioral: lower serum hippuric acid was associated with higher depression scores.	[73]
Human, *n* = 128. Severe AUD patients hospitalized for 3-week detoxification, *n* = 96; HC *n* = 32, age 47 ± 10 y.	Cross-sectional: sAUD vs. HC; longitudinal: T1 (admission) vs. T2 (day18–19 abstinence) within sAUD. Exposure: Chronic heavy; sAUD: mean 145 ± 94 g/day, drinking duration 16 ± 11 years	Self-report (TLFB, DSM criteria).	FM: not assessed. Met: plasma; untargeted LC-MS (reverse phase + HILIC, ± polarity).	N/A	sAUD vs. HC: ↑ long-chain FAs, bile acids, 3-hydroxyvaleric acid, cortisol; ↓ LPCs (odd-chain, ether-bond), furan FAs, xanthines (paraxanthine, theobromine, theophylline), microbial metabolites (indole-3-propionic acid, p-cresol sulfate, hippuric acid, pyrocatechol sulfate). After 3-week abstinence: ↑ xanthines, indole derivatives, hippuric acid, pyrocatechol sulfate, odd-chain/ether LPCs; ↓ 3-hydroxyvaleric acid, C16 acylcarnitines. Correlations: xanthines, pyrocatechol sulfate, hippuric acid negatively correlated with anxiety/depression/craving. Brain validation: metabolites detected in frontal cortex and CSF.	Behavioral: hippuric acid, pyrocatechol sulfate, and xanthines (paraxanthine, theobromine, theophylline) negatively correlated with depression, anxiety, and alcohol craving. Brain validation: these metabolites detected in frontal cortex and CSF postmortem of heavy alcohol users.	[79]
Human, AD *n* = 20–90, and HC *n* = 10–16, 48 ± 10 y.	Cross-sectional: AD vs. HC; longitudinal: T1 (admission) vs. T2 (day 19 abstinence) within AD. Exposure: Chronic heavy; AD: ≥23 g/day (mean 180 g/day); HC: <20 g/day.	Self-report (DSM criteria, TLFB) + fecal ethanol by GC/MS.	FM: not assessed (data from Leclercq et al., 2014 [39]). Met: feces (GC/MS for ethanol) and plasma (BHB, NEFA assays)	N/A (same cohort as Leclercq et al., 2014 [39]).	AD vs. HC: ↑ fecal ethanol.	Behavioral: BHB positively correlated with sociability and negatively with depression and craving. Physiological (neuroimaging): BHB positively correlated with white matter integrity (fractional anisotropy in forceps major and left superior longitudinal fasciculus).	[74]

Note: The abbreviations used in this table are defined below: 5-HIAA, 5-hydroxyindoleacetic acid; AB, abstinent individuals; AD, alcohol-dependent; ADS, Alcohol Dependence Scale; AUD, Alcohol Use Disorder; AUDIT, Alcohol Use Disorders Identification Test; CD, current drinkers; CMP, cytidine monophosphate; CSR, computerized self-administration reinforcement; CT, control; DCA, deoxycholic acid; FA, fatty acids; FM, fecal microbiota; G1P, glucose-1-phosphate; GC-FID, gas chromatography with flame ionization detection; GP, general population; HC, healthy controls; h, hours; hSPME, headspace solid-phase microextraction; IP, intestinal permeability; IQR, interquartile range; L, long-term drinking; LCA, lithocholic acid; LPC, lysophosphatidylcholine; LPS, lipopolysaccharide; MDD, major depressive disorder; Met, metabolomics; MNA, Mini Nutritional Assessment; N/A, Not Applicable; NEFA, non-esterified fatty acids; NHP, non-human primate; S, short-term drinking; T1/T2, time point 1/time point 2; TLFB, Timeline Follow-back; TMP, thiamine monophosphate; UMP, uridine monophosphate; UPLC-MS/MS, ultra-performance liquid chromatography–tandem mass spectrometry; V, variable region of the 16S rRNA gene; *w*/*v*, weight/volume; XMP, xanthosine monophosphate; ↓, decrease; ↑, increase.

### 3.2. Quality of Included Studies

The methodological quality and risk of bias of the included studies were assessed using two complementary tools, selected according to the study design and population. For studies involving non-human primates (NHPs), the SYRCLE Risk of Bias tool was applied [80], specifically designed for animal intervention studies. This tool examines 10 domains, including sequence generation, blinding, and incomplete outcome data, classifying the risk as low, uncertain, or high. For observational studies in humans, the Newcastle–Ottawa Scale (NOS) was used. The NOS assesses three main domains: participant selection, comparability of groups, and exposure/outcome determination, with a maximum score of 9 points [81]. All assessments were performed independently by two reviewers, and disagreements were resolved by consensus.

**Figure 2:**
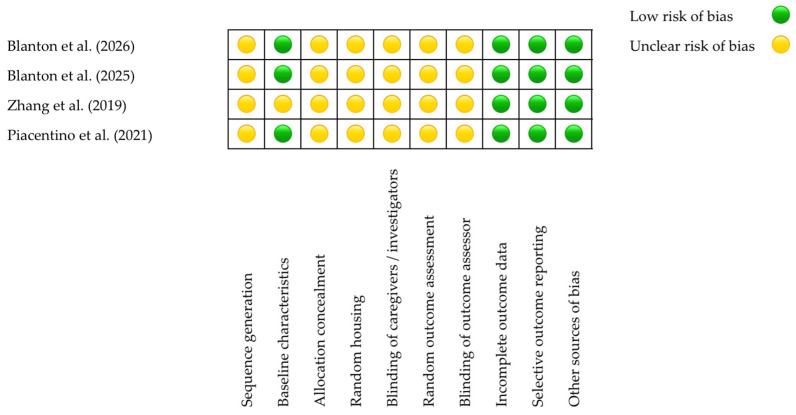
SYRCLE Risk of Bias tool [65,70,71,72].

The four studies with non-human primates [65,70,71,72] showed a consistent pattern (Figure 2). A low risk of bias was observed in the domains of “Incomplete outcome data” and “Selective outcome reporting” in all studies (100%). “Baseline characteristics” were rated as low in three studies (75%) due to longitudinal designs [65,71,72] that used animals as their own controls. Conversely, unclear risk was found in 100% of the studies for “Sequence Generation”, “Allocation Concealment”, “Randomized Housing”, “Caregiver/Investigator Blinding”, “Randomized Outcome Assessment”, and “Outcome Assessor Blinding”, reflecting insufficient information on randomization and blinding procedures. No study was rated as high risk in any domain.

The NOS results from the human studies (Table 2) showed scores ranging from 5 to 9 (median: 8). Six studies (75%) were classified as high quality (NOS ≥ 7) [39,74,75,77,78,79]. Two studies (25%) were rated as moderate quality (NOS 5–6) [73,76]. No studies were classified as low quality. Strengths of the human studies included adequate participant selection (mean selection score: 3.5/4) and objective outcome assessment (mean outcome score: 2.7/3), and 7/8 studies used validated instruments for alcohol exposure (TLFB or DSM criteria). The main methodological challenge was limited comparability (mean comparability score: 1.5/2), as only 4/8 studies performed comprehensive statistical adjustments for multiple confounding factors. None of the included human studies reported blood alcohol concentration or other objective biomarkers of recent alcohol intake.

In summary, the included evidence is of moderate to high quality. Human studies demonstrate robust outcome selection and assessment but have limitations regarding comparability and reliance on self-reported exposure. Non-human primate studies show high data comprehensiveness but lack information on randomization and blinding.

### 3.3. Findings from Non-Human Primate Studies

Four studies investigated the effects of alcohol exposure on gut microbiota composition and microbiota-derived metabolites in non-human primates, including rhesus macaques (*n* = 3) and baboons (*n* = 1). The duration of alcohol exposure ranged from three months to more than twelve years, and two studies additionally evaluated the effects of a short (3–5 days) abstinence period.

Alcohol consumption was associated with alterations in gut microbial diversity and taxonomic composition. Using a rhesus macaque model that included both adolescent animals exposed to alcohol for three months and adult animals with a history of approximately five years of alcohol self-administration, Zhang et al. [70] reported reduced α-diversity and marked shifts in microbial community structure during alcohol exposure. These changes included an increased relative abundance of Firmicutes, particularly the family Streptococcaceae, together with decreased abundance of Bacteroidetes and several alcohol-sensitive taxa, including Euryarchaeota, Tenericutes, Lentisphaerae, and Verrucomicrobia. 

In baboons, Piacentino et al. [65] observed significantly lower α-diversity and altered β-diversity in animals with long-term excessive alcohol consumption (median 12.1 years) compared with short-term drinkers (median 2.7 years) and controls. In contrast, no significant differences in α-diversity were observed between short-term drinkers and controls. At the family level, Lactobacillaceae and Streptococcaceae were enriched in long-term drinkers, whereas Prevotellaceae and Lachnospiraceae were reduced. Consistent with these findings, long-term alcohol exposure was associated with increased abundances of the genera *Lactobacillus* and *Streptococcus*, and decreased abundances of *Prevotella*, *Blautia*, *Dialister*, and *Sutterella.* In contrast, short-term drinkers showed enrichment of *Faecalibacterium*, *Parabacteroides*, *Oribacterium*, *Collinsella*, *Desulfovibrio*, and *Eubacterium*. Control animals exhibited higher abundances of *Butyrivibrio*, *Dorea*, and *Ruminococcus*.

Similarly, in a study conducted in macaques before and after 6 months of ethanol consumption, Blanton et al. [71] reported significant alcohol-related alterations in β-diversity, although no differences were observed in the number of amplicon sequence variants (ASVs). Significant differences in microbial composition and abundance were also identified when sex was included as a biological variable. Alcohol consumption reduced the relative abundance of several SCFA-producing taxa, including the genera *Eubacterium* and *Subdoligranulum* and members of the family Butyricoccaceae, while increasing the abundance of bacteria associated with immunoregulatory functions, such as *Prevotella*, *Faecalibacterium*, *Dorea*, and *Sutterella*. In addition, the abundance of several bacterial taxa showed significant dose-dependent associations with ethanol intake, including positive correlations for *Lactobacillus*, *Terrisporobacter*, and *Colidextribacter*, and negative correlations for *Clostridium*, *Catenibacterium*, *Sarcina*, and members of the family Oscillospiraceae.

In a subsequent study, Blanton et al. [72] identified significant alterations in gut microbial community composition after 12 months of voluntary alcohol self-administration in rhesus macaques, as demonstrated by both weighted and unweighted principal coordinate analysis (PCoA), although no differences were observed in the number of ASVs. Alcohol exposure was associated with reduced relative abundances of several SCFA-producing taxa, including members of the families Prevotellaceae, Muribaculaceae, Rikenellaceae, and Christensenellaceae, as well as taxa belonging to the order Bacteroidales and the class Clostridia. Conversely, increased abundances of *Colidextribacter*, *Succinivibrio*, *Eubacterium*, and *Monoglobus* were observed. Correlation analyses further revealed positive associations between blood alcohol levels (BAL) and the abundance of *Subdoligranulum*, whereas *Anaerosporobacter*, members of the family Selenomonadaceae, *Fibrobacter*, and Bradymonadales were negatively associated with BAL.

Substantial alterations in metabolomic profiles were also reported. Piacentino et al. [65] identified 63 metabolites showing a significant group × condition interaction (FDR-adjusted *p* < 0.01) among the 534 metabolites analyzed. Approximately two-thirds of these metabolites belonged to the amino acid (36.5%) and lipid (30.1%) super pathways. Altered amino acid metabolism involved aromatic amino acids (tyrosine, phenylalanine, and tryptophan), branched-chain amino acids (leucine, isoleucine, and valine), and sulfur-containing amino acids (methionine, cysteine, homocysteine, and taurine). Lipid-related alterations primarily affected fatty acids and their derivatives, phospholipid metabolites, lysophospholipids, phosphatidylethanolamines, cholesterol derivatives, and endocannabinoids. Additional changes were observed in pentose metabolism, amino sugar metabolism, nucleotide metabolism, and tricarboxylic acid cycle (TCA) intermediates. In contrast, although Zhang et al. [70] identified 410 biochemical compounds, no metabolites differed significantly between alcohol-consuming and control animals after correction for multiple testing.

Blanton et al. [71] observed increased fecal concentrations of mercaptopyruvic acid, sulfuric acid, and urocanic acid, together with decreased concentrations of tyrosine, N-methylproline, Gly-Pro, and several medium- and long-chain fatty acids, including dodecanoic, palmitic, oleic, stearic, nonadecanoic, behenic, and lignoceric acids. In contrast, heptanoic acid was increased following six months of alcohol exposure. Plasma metabolomic analyses additionally revealed increased concentrations of acetyltaurine, β-hydroxybutyric acid, stachydrine, urate, kynurenate, guanine, guanosine, and glutamic acid.

Likewise, Blanton et al. [72] reported a distinct separation of the fecal metabolome after 12 months of alcohol consumption. Fecal analyses revealed increased concentrations of lithocholic acid, proline, quinolinate, glucose-1-phosphate, and L-carnitine, together with reduced concentrations of thiamine monophosphate, xanthosine monophosphate, uridine monophosphate, and cytidine monophosphate. In addition, alcohol exposure was associated with decreased levels of several medium- and long-chain fatty acids, including palmitic, oleic, stearic, 11-eicosenoic, nonadecanoic, dodecanoic, and behenic acids. Plasma metabolomic analyses also showed a clear separation between alcohol-exposed and control animals, with increased concentrations of glycochenodeoxycholate, isoleucine, phenylalanine, proline, tyrosine, and arginine, together with decreased levels of pipecolate, cortisol, and corticosterone.

Alterations in SCFAs were specifically evaluated in two studies. Blanton et al. [71] reported a significant reduction in fecal propionic acid following six months of alcohol exposure. Extending these findings, Blanton et al. [72] reported significant decreases in multiple fecal SCFAs after 12 months of alcohol consumption, including acetate, propionate, isobutyrate, *n*-butyrate, 2-methylbutyrate, isovalerate, and *n*-valerate.

Two studies assessed the effects of abstinence. Zhang et al. [70] reported partial recovery of several alcohol-associated microbial alterations following five days of abstinence, including changes in the families Streptococcaceae and Bacteroidaceae, together with significant changes in 22 metabolites (FDR < 0.05), more than one third of which were fatty acids. Glucose-6-phosphate and fructose-6-phosphate, part of energy utilization upstream of the Krebs cycle, were increased following abstinence. Glycolysis-related compounds were also overrepresented among the significantly altered metabolites, although this enrichment did not remain significant after correction for multiple testing. The metabolomic response to abstinence showed greater variability in younger animals than in adults. In baboons, Piacentino et al. [65] found no significant changes in α-diversity between drinking and abstinence (3-day) conditions, and only minor alterations in microbial composition, with a limited number of taxa differing between conditions. However, principal components analysis (PCA) showed a clear separation of long-term drinkers from short-term drinkers and controls, as well as a separation between conditions within the long-term group. In contrast, drinking and abstinence conditions clustered together in the short-term drinking and control groups, which displayed a closer metabolic profile.

### 3.4. Findings from Human Studies

#### 3.4.1. Gut Microbiota and Microbial Metabolite Alterations Associated with Alcohol Consumption

The findings were organized into two complementary sections: (i) alcohol-associated alterations in gut microbiota composition and (ii) alcohol-associated alterations in gut microbiota-derived metabolites. Four studies reported changes in gut microbiota composition, while seven studies examined microbial metabolites or microbiota-related metabolic profiles. As several investigations assessed both microbial communities and metabolomic profiles, some studies are presented in both sections.

##### Alcohol-Associated Alterations in Gut Microbiota Composition

Leclercq et al. [39] investigated gut microbiota composition in alcohol-dependent (AD) individuals stratified according to intestinal permeability (IP). Although no significant differences were observed at the phylum level, AD subjects with increased IP (high IP) displayed a distinct microbial profile compared with both controls and AD subjects with normal IP (low IP). At the family level, high-IP subjects showed reduced abundances of Ruminococcaceae and Incertae Sedis XIII together with increased abundances of Lachnospiraceae and Incertae Sedis XIV. At the genus level, decreased abundances of *Ruminococcus*, *Faecalibacterium*, *Subdoligranulum*, *Oscillibacter*, and *Anaerofilum* were observed, whereas *Dorea*, *Blautia*, and *Megasphaera* were enriched. Quantitative PCR analyses further revealed lower total bacterial counts, a marked depletion of *Faecalibacterium prausnitzii*, and reduced levels of *Bifidobacterium* spp. in high-IP subjects. In contrast, the microbial profiles of low-IP AD subjects closely resembled those of controls. Extending these findings, Leclercq et al. [74], using the same cohort of AD individuals previously characterized, reported that increased intestinal permeability was associated with lower sociability scores.

Among individuals with chronic alcohol overconsumption and controls, Bjørkhaug et al. [76] found no significant differences in gut microbial α-diversity or in the Firmicutes/Bacteroidetes ratio. However, β-diversity analyses revealed significant differences in microbial community composition between groups (weighted UniFrac, *p* = 0.003; unweighted UniFrac, *p* = 0.002), with a subset of alcohol overconsumers displaying a distinct microbial profile. At the phylum level, alcohol overconsumers showed a higher relative abundance of Proteobacteria, whereas no significant differences were observed for Firmicutes, Bacteroidetes, or Actinobacteria. At the genus level, a lower relative abundance of *Faecalibacterium* and higher relative abundances of *Sutterella*, *Clostridium*, and *Holdemania* were identified in alcohol overconsumers. Additional LEfSe analyses confirmed the enrichment of Proteobacteria and further identified lower abundances of Ruminococcaceae and *Prevotella* in alcohol overconsumers, together with increased representation of Enterobacteriaceae and Desulfovibrionaceae and reduced abundance of several taxa within the class Clostridia.

More recently, Piacentino et al. [78] further investigated gut microbiota composition in AUD. In a cohort including actively drinking individuals with AUD, abstinent individuals with AUD, and healthy controls, the authors reported no significant differences in gut microbial β-diversity between groups and no differences in Shannon or Simpson diversity indices. However, significant group differences were observed for microbial richness, as assessed by Observed ASVs and Chao1 indices, with the lowest richness detected in abstinent individuals. Comparisons between actively drinking individuals and healthy controls revealed only limited taxonomic differences, with *Dorea* and *Lachnospira* identified as differentially abundant between groups. The most pronounced microbiota alterations were observed in abstinent individuals and are described in Section 3.4.2.

Similarly, Wang et al. [75] observed compositional differences in the gut microbiota of alcohol-exposed individuals despite limited changes in microbial richness. In a cohort of long-term alcohol-exposed individuals and non-drinking controls, these authors reported no significant differences in gut microbial alpha diversity as assessed by the Chao1 richness index. However, beta-diversity analysis revealed a significant shift in overall microbial community structure among alcohol-exposed participants. Metagenomic profiling showed that the gut microbiota of controls was predominantly characterized by *F. prausnitzii* C and *F. prausnitzii* G populations, whereas alcohol-exposed individuals were characterized by a predominance of *F. prausnitzii* E and *Enterocloster bolteae*. These findings suggest that chronic alcohol consumption is associated with compositional alterations of the gut microbiota rather than major changes in microbial richness.

Overall, the available evidence suggests that alcohol consumption may be more consistently associated with shifts in gut microbial composition and specific taxonomic groups than with marked reductions in global microbial diversity.

##### Alcohol-Associated Alterations in Gut Microbiota-Derived Metabolites

Studies investigating gut microbiota-derived metabolites revealed alcohol-associated alterations across multiple biological matrices, including fecal volatile organic compounds (VOCs), untargeted fecal metabolomic profiles, and circulating metabolites.

Leclercq et al. [39] investigated fecal VOCs in AD individuals stratified according to IP. Metabolic profiling revealed distinct VOC signatures in AD subjects with high IP compared with those with low IP. Among the discriminating metabolites, phenolic and indolic compounds derived from aromatic amino acid metabolism were associated with gut-barrier status. Specifically, phenol was markedly increased in high-IP subjects, whereas 4-methyl phenol (also called *p*-cresol) was more abundant in low-IP subjects. In addition, indole and 3-methyl indole were abundant in low-IP subjects but were markedly reduced or nearly absent in high-IP individuals. More broadly, several VOCs, including 2-methyl-1-butanol and methanethiol, were detected in controls but not in AD subjects, whereas compounds belonging to the alcohol, alkane, and benzene classes were identified exclusively in AD individuals.

Additional evidence of alcohol-associated alterations in the fecal volatile metabolome was provided by Couch et al. [77]. Using GC–MS-based profiling of fecal VOCs, the authors identified a distinct fecal metabolomic signature in alcoholic participants compared with healthy controls. Multivariate analyses, including PCA and hierarchical clustering analyses, consistently discriminated alcoholic and control samples, indicating substantial alterations in microbial metabolic activity. Among metabolites potentially related to gut microbial metabolism, reduced levels of the SCFAs propionate and isobutyrate were observed in alcoholic participants. Additional metabolic changes included increased tetradecane and decreased levels of several fatty alcohols, as well as reduced concentrations of caryophyllene, camphene, dimethyl disulfide, and dimethyl trisulfide. Together, these findings suggest that chronic alcohol consumption is associated with broad alterations in the fecal volatile metabolome, including changes in metabolites linked to microbial fermentation, oxidative stress, and intestinal barrier function. The authors proposed that some of these VOCs could serve as candidate biomarkers of alcohol-related gut dysfunction and chronic alcohol exposure. Notably, similar discrimination between alcoholic and control groups was observed in both endoscopically collected and home-collected fecal samples, supporting the potential utility of fecal VOC profiling as a non-invasive approach for biomarker discovery.

Beyond VOCs, subsequent research examined specific microbial fermentation products, particularly SCFAs. In individuals with chronic alcohol overconsumption, Bjørkhaug et al. [76] reported a significantly lower relative proportion of fecal butyric acid compared with controls (11.9% vs. 16.3%, *p* = 0.012), together with a trend toward lower absolute concentrations. No significant differences were observed for other SCFAs or for total SCFA concentrations. Notably, Proteobacteria abundance was inversely correlated with butyric acid, acetic acid, valeric acid, and total SCFA levels in alcohol overconsumers, whereas *Faecalibacterium* abundance was positively associated with butyric acid concentrations.

More comprehensive metabolomic approaches further explored alcohol-associated alterations in the fecal metabolome. Using untargeted fecal metabolomic profiling, Piacentino et al. [78] identified only limited metabolite differences between actively drinking individuals with AUD and healthy controls. Five metabolites were differentially abundant between groups, including lower concentrations of aconitate and phytosphingosine, and higher concentrations of 3-hydroxypalmitoylcarnitine, oleoylcarnitine, and 1-methylxanthine in actively drinking individuals. Overall, the most substantial metabolomic alterations were observed in abstinent individuals, who accounted for most of the differentially abundant metabolites identified in the study. These abstinence-related findings are described in Section 3.4.2.

Whereas Piacentino et al. [78] identified only limited metabolomic differences between actively drinking individuals and controls, Wang et al. [75] reported more extensive alterations in the fecal metabolome associated with long-term alcohol exposure. The authors performed untargeted fecal metabolomic analyses in long-term alcohol-exposed individuals and non-drinking controls and identified marked differences in metabolite composition between groups. Multivariate analysis (PLS-DA) demonstrated a clear separation of the metabolomic profiles, with 79 differential metabolites detected (48 increased and 31 decreased in alcohol-exposed participants). The metabolites showing the strongest differences included L-arogenate, adenosine, and hyocholate, while pathway enrichment analysis revealed alterations in riboflavin metabolism, steroid biosynthesis, branched-chain amino acid biosynthesis, D-amino acid metabolism, and purine metabolism. Notably, adenosine emerged as a key metabolite associated with chronic alcohol exposure and was identified as the only metabolite consistently altered across the human gut metabolome and both gut and serum metabolomes in alcohol-exposed mice, leading the authors to propose purine metabolism and adenosine signaling as potential mechanistic mediators of the reduced anesthetic efficacy associated with chronic alcohol exposure.

In addition to fecal metabolomic alterations, several studies identified alcohol-associated changes in circulating metabolites potentially linked to gut microbial activity. Using untargeted plasma metabolomics, Leclercq et al. [79] identified a distinct metabolic profile in patients with severe AUD compared with healthy controls. Among the metabolites potentially linked to gut microbial metabolism, circulating levels of hippuric acid, indole-3-propionic acid, p-cresol sulfate, pipecolic acid, and pyrocatechol sulfate were significantly reduced in patients with AUD. In contrast, 3-hydroxyvaleric acid was increased. Furthermore, alcohol intake was negatively correlated with hippuric acid, p-cresol sulfate, pyrocatechol sulfate, and indole-3-propionic acid, suggesting that these compounds are particularly sensitive to alcohol exposure. Several of these metabolites, including hippuric acid and pyrocatechol sulfate, were also negatively associated with the severity of depression, anxiety, and alcohol craving.

Beyond metabolites linked to microbial activity, the metabolomic profile of AUD patients was characterized by marked alterations in lipid species, acylcarnitines, bile acids, steroid-related metabolites, and several xanthine metabolites. Furthermore, targeted analyses of postmortem cerebrospinal fluid and frontal cortex samples, obtained from individuals with a history of heavy alcohol use and controls, confirmed the presence of several alcohol-sensitive metabolites, including hippuric acid, pyrocatechol sulfate, paraxanthine, theobromine, and 3-hydroxyvaleric acid, supporting their potential relevance within the CNS [79].

Notably, the association between alcohol exposure and reduced circulating hippuric acid levels was further supported by Kärkkäinen et al. [73]. Although not exclusively microbiota-derived, hippuric acid is considered a microbiota–host co-metabolite because its biosynthesis depends on both microbial transformation of dietary phenolic compounds and subsequent hepatic metabolism. Using targeted serum metabolomics, Kärkkäinen et al. [73] examined associations between self-reported alcohol consumption and circulating metabolite concentrations in a cohort of non-depressed individuals from the general population. Alcohol use was negatively associated with serum hippuric acid concentrations and positively associated with chenodeoxycholic acid levels. After correction for multiple testing, these were the only metabolite associations that remained statistically significant in the general population cohort. Notably, individuals reporting moderate alcohol consumption (≥6 drinks per week) exhibited hippuric acid concentrations comparable to those observed in patients with major depressive disorder, irrespective of alcohol intake within the depressed cohort. Additional nominal associations were observed for several metabolites, including lower concentrations of glutamine, serine and 5-hydroxyindoleacetic acid, and higher concentrations of taurocholic acid and cytidine, although these findings did not survive correction for multiple comparisons. These findings suggest that even moderate alcohol consumption may be associated with metabolomic alterations that overlap with those observed in depression, particularly reduced circulating hippuric acid levels, supporting a potential role for gut microbiota-related metabolic pathways in the relationship between alcohol use and depressive symptoms.

Overall, the available evidence indicates that alcohol consumption is associated with alterations in both fecal and circulating metabolite profiles, affecting microbial fermentation products, aromatic amino acid metabolites, purine-related compounds, and microbiota–host co-metabolites. Several of these metabolites were consistently linked to alcohol exposure across independent cohorts and may represent functionally relevant markers of alcohol-associated gut dysbiosis.

#### 3.4.2. Effects of Alcohol Abstinence

The duration of abstinence varied across human studies, ranging from approximately 3 weeks [39,74,79] to more than 6 weeks [78]. This variability should be considered when comparing the extent of microbiota and metabolomic recovery reported by different studies. These studies consistently indicated that withdrawal is accompanied by microbiota and metabolomic changes, although recovery toward control profiles appeared to be incomplete and heterogeneous across studies.

In AD individuals stratified according to IP, Leclercq et al. [39] reported that three weeks of abstinence were associated with a partial recovery of gut microbial alterations in subjects with high IP. Ruminococcaceae increased significantly, whereas *Ruminococcus* and *Subdoligranulum* showed non-significant increases. Quantitative PCR analyses further demonstrated recovery of the total amount of bacteria together with increased abundances of *Bifidobacterium* spp. and *Lactobacillus* spp., reaching levels comparable to those of controls. In contrast, *F. prausnitzii* remained markedly depleted following abstinence. Among the VOCs evaluated, 4-methyl phenol showed increased levels following abstinence in subjects with high IP. Notably, despite normalization of intestinal permeability, subjects with initially high IP continued to exhibit elevated levels of depression, anxiety, and alcohol craving.

Despite evidence of partial microbiota recovery during abstinence, other findings suggested that some alcohol-associated metabolic alterations may persist after alcohol withdrawal. In detoxified alcohol-dependent individuals, Leclercq et al. [74] reported that fecal ethanol remained detectable in 19 of 20 participants after three weeks of abstinence and was present at higher levels than in controls, suggesting persistent endogenous microbial ethanol production despite alcohol withdrawal. No additional analyses of gut microbiota composition were performed. The authors also evaluated plasma β-hydroxybutyrate (BHB) and non-esterified fatty acids (NEFAs) and examined their associations with psychological symptoms and neuroimaging measures; these findings are considered in the Section 4 because they reflect host metabolic pathways potentially influenced by the gut microbiota rather than directly microbiota-derived metabolites.

Further evidence indicated that abstinence may be associated with a distinct microbial and metabolomic profile rather than a complete return to the patterns observed in healthy controls. In the study conducted by Piacentino et al. [78], abstinent individuals (≥6 weeks) with AUD exhibited the most pronounced microbiota and metabolite alterations. Although no differences in β-diversity or Shannon and Simpson diversity indices were observed, abstinent individuals showed reduced microbial richness and distinct taxonomic profiles compared with both actively drinking individuals and healthy controls. Specifically, higher relative abundances of *Streptococcus*, *Eisenbergiella*, and *Candidatus Stoquefichus* were observed compared to actively drinking individuals, whereas differences involving *Streptococcus*, *Fusicatenibacter*, *Lachnospira*, *Lachnospiraceae_UCG_001*, and *Roseburia* distinguished abstinent individuals from healthy controls. Untargeted fecal metabolomic profiling revealed a distinct clustering of abstinent individuals and identified widespread alterations in lipid metabolites, together with lower concentrations of the tryptophan-derived metabolites skatol and xanthurenate, and higher concentrations of fructosyllysine, cystine, carboxymethylarginine, 1-methylxanthine, and N6-carboxymethyllysine compared with both actively drinking individuals and healthy controls.

Complementing these observations, longitudinal metabolomic analyses provided additional insight into metabolic changes occurring during early alcohol withdrawal. In a longitudinal analysis of 96 patients with severe AUD undergoing a 3-week detoxification program, Leclercq et al. [79] observed marked metabolomic changes during alcohol withdrawal. Among microbiota-related metabolites, circulating levels of the tryptophan derivatives indole-3-propionic acid and indole-3-acetic acid increased during abstinence, whereas 3-hydroxyvaleric acid decreased. Beyond these microbiota-related metabolites, several other metabolic alterations were observed, including increases in xanthine metabolites (paraxanthine, theobromine, and theophylline), odd-chain and ether-linked lysophosphatidylcholines, and decreases in acylcarnitines and lysophosphatidylcholines containing 16- or 18-carbon fatty acid tails. Overall, alcohol withdrawal was associated with a broad reorganization of the circulating metabolome, with many metabolites changing in the opposite direction to that observed at baseline.

Taken together, these findings suggest that alcohol abstinence is accompanied by substantial microbiota and metabolomic remodeling. Although some alterations appear to move toward control levels during withdrawal, other microbial and metabolic features persist or remain distinct, indicating that recovery following alcohol cessation may be only partial during the early stages of abstinence.

## 4. Discussion

The present systematic review aimed to synthesize current evidence on the effects of alcohol consumption and abstinence on gut microbiota-derived metabolites and their relationship with alterations in gut microbiota composition in humans and non-human primates. Overall, the available literature indicates that chronic alcohol exposure is associated with alterations in both gut microbiota composition and gut microbiota-related metabolic pathways. Although the specific microbial taxa affected varied considerably between studies, several investigations consistently identified alcohol-associated changes in microbial metabolic outputs, including SCFAs, tryptophan-derived metabolites, phenolic compounds, bile acids, and purine-related metabolites [39,73,75,76,77,79]. In contrast, findings regarding global microbial diversity were less consistent. Significant alterations in β-diversity were reported in some cohorts [75,76], whereas α-diversity often remained unchanged [75,76,78], and reductions in richness were observed only in specific populations, such as abstinent individuals with AUD [78]. Taken together, these observations suggest that alcohol consumption may exert a more consistent impact on microbial metabolic function than on overall microbial diversity, highlighting the importance of integrating metabolomic approaches into investigations of alcohol-related gut dysbiosis.

**Figure 3:**
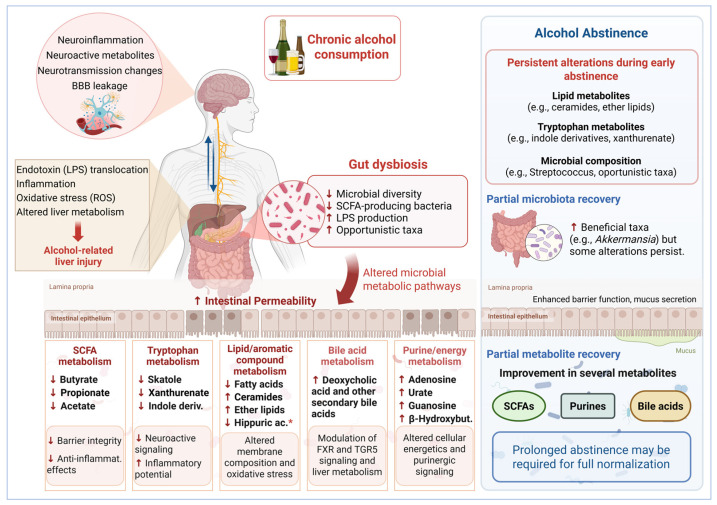
Schematic overview of alcohol-induced gut microbiota dysfunction and microbiota-derived metabolic alterations across the gut–liver–brain axis. The diagram summarizes the principal mechanisms and recurrent findings identified across the studies included in this systematic review. Chronic alcohol consumption promotes gut dysbiosis, characterized by reduced microbial diversity, depletion of SCFA-producing bacteria, increased LPS-producing and opportunistic taxa, and increased intestinal permeability. These alterations are accompanied by disruptions in microbiota-associated metabolic pathways, with the most consistent findings across both humans and non-human primates involving SCFA metabolism, tryptophan metabolism, and hippuric acid-related metabolism, together with recurrent disturbances in lipid, bile acid, and purine/energy metabolism. Collectively, these alterations may contribute to intestinal barrier dysfunction, immune dysregulation, liver injury, neuroinflammation, and altered gut–brain communication. Alcohol abstinence is associated with only partial recovery of the gut microbiota and metabolome during the early stages of withdrawal, whereas prolonged abstinence may be required for more complete normalization. * Hippuric acid is a host–microbial co-metabolite generated through hepatic conjugation of microbiota-derived benzoate. Created in BioRender. Echeverry-Alzate, V. (2026) https://BioRender.com/pyu2auo (accessed on 23 July 2026). SCFAs, short-chain fatty acids; ↓, decrease; ↑, increase.

Despite the heterogeneity observed across studies, several recurrent microbial alterations emerged. Alcohol exposure was repeatedly associated with shifts in microbial community composition, whereas changes in global diversity metrics were less consistent. In both human and non-human primate studies, alcohol consumption was associated with reduced abundances of taxa commonly linked to intestinal homeostasis, including *Faecalibacterium*, members of the family Ruminococcaceae, and other butyrate-producing bacteria [39,70,71,76]. Conversely, increases in Proteobacteria and other taxa frequently associated with dysbiosis and inflammatory conditions were observed, most notably in the cohort studied by Bjørkhaug et al. [76]. However, the specific microbial signatures varied substantially across studies, likely reflecting differences in alcohol exposure patterns, host characteristics, analytical methodologies, and biological matrices. These findings suggest that alcohol-related dysbiosis may be characterized less by disruption of a specific taxonomic profile and more by alterations in the ecological balance and functional capacity of the gut microbiota (Figure 3). This interpretation is consistent with the observation that metabolomic changes were generally more reproducible than taxonomic alterations across studies, supporting the concept that distinct microbial communities may converge toward similar functional outputs [82].

The metabolomic findings further suggest that alcohol-associated dysbiosis may have functional consequences for microbial fermentation processes. Several studies identified alterations in SCFAs, particularly butyrate, propionate, and isobutyrate, which are key products of bacterial fermentation and play important roles in maintaining intestinal barrier integrity, regulating immune responses, and mediating gut–brain communication [51,52,53]. In humans, reduced fecal butyrate was reported in individuals with chronic alcohol overconsumption [76], while lower levels of propionate and isobutyrate were observed in alcoholic participants by Couch et al. [77]. Similar reductions in SCFAs were also reported in non-human primate models of chronic alcohol exposure [71,72]. Although the specific SCFAs affected varied across studies, the overall pattern suggests that alcohol consumption may impair microbial fermentation capacity. This interpretation is further supported by the recurrent depletion of microbial taxa commonly involved in SCFA production, including *Faecalibacterium* and members of the family Ruminococcaceae. Given the established role of SCFAs in preserving epithelial barrier function and limiting intestinal inflammation [83,84], reduced SCFA availability may represent one potential mechanism linking alcohol-induced dysbiosis to the increased IP and systemic inflammation frequently observed in individuals with chronic alcohol exposure [85].

Beyond SCFAs, alterations in tryptophan metabolism emerged as another recurrent finding across the studies included in this review. Several investigations identified alcohol-associated changes in indole-related metabolites derived from microbial tryptophan metabolism, including indole, 3-methylindole (skatol), indole-3-propionic acid, indole-3-acetic acid, xanthurenate, and other compounds within the tryptophan metabolic pathway [39,71,78,79]. Notably, Leclercq et al. [39] reported markedly reduced levels of indole and 3-methylindole in AD individuals with increased IP, whereas Leclercq et al. [79] observed reduced circulating concentrations of indole-3-propionic acid in patients with severe AUD and a subsequent increase during abstinence. Similarly, Piacentino et al. [78] found lower concentrations of skatol and xanthurenate in abstinent individuals with AUD compared with both actively drinking participants and healthy controls. Although the specific metabolites differed among studies, these findings collectively indicate that alcohol exposure is associated with disruptions in microbial tryptophan metabolism that may persist beyond active drinking. This observation is potentially relevant because microbial tryptophan metabolites contribute to intestinal homeostasis through activation of the AhR, a signaling pathway involved in epithelial barrier maintenance, mucosal immunity, and host–microbiota interactions [55]. Consequently, disturbances in microbial tryptophan metabolism may represent an additional mechanism through which alcohol-induced dysbiosis contributes to intestinal dysfunction and altered host physiological responses [85,86,87].

In addition to SCFAs and tryptophan-derived compounds, several studies identified alterations in microbiota-related metabolites originating from the microbial metabolism of dietary phenolic compounds and aromatic amino acids. Among these, hippuric acid emerged as one of the most consistently affected metabolites. Both Leclercq et al. [79] and Kärkkäinen et al. [73] reported reduced circulating hippuric acid concentrations associated with alcohol exposure, while Leclercq et al. [79] also observed reductions in other microbial metabolites, including p-cresol sulfate, pyrocatechol sulfate, and pipecolic acid. Furthermore, alcohol intake was negatively correlated with hippuric acid, p-cresol sulfate, pyrocatechol sulfate, and indole-3-propionic acid concentrations, suggesting that these metabolites are particularly sensitive to alcohol exposure. Importantly, several of these compounds were also associated with clinical outcomes. Hippuric acid and pyrocatechol sulfate were negatively correlated with depression, anxiety, and alcohol craving in patients with severe AUD [79], while Kärkkäinen et al. [73] found that reduced hippuric acid concentrations were associated with both alcohol consumption and depressive symptom severity. Although causality cannot be inferred from these observations, the convergence of findings across independent cohorts suggests that microbiota-related metabolic alterations may contribute to the biological pathways linking alcohol exposure to neuropsychiatric manifestations. This interpretation is further supported by the fact that hippuric acid is considered a microbiota–host co-metabolite whose biosynthesis depends on both microbial transformation of dietary phenolic compounds and subsequent hepatic metabolism [88,89]. Consequently, alterations in circulating hippuric acid may reflect disturbances affecting both gut microbial activity and host metabolic processing. More broadly, these findings are consistent with growing evidence indicating that gut microbiota-derived metabolites participate in bidirectional communication between the gut and the CNS and may influence behavior, cognition, and emotional regulation [90,91].

Beyond microbial fermentation products and aromatic amino acid metabolites, alcohol exposure was also associated with alterations in bile acid metabolism. Leclercq et al. [79] reported increased levels of several bile acids in patients with severe AUD, while Kärkkäinen et al. [73] identified a positive association between alcohol consumption and circulating chenodeoxycholic acid concentrations. Similarly, Wang et al. [75] identified hyocholate among the metabolites showing the strongest differences between alcohol-exposed individuals and controls. Although the specific bile acids varied across studies, these findings collectively suggest that alcohol consumption may influence pathways involved in bile acid synthesis, transformation, and circulation. Such alterations are biologically plausible given the bidirectional interactions between gut microbiota and bile acid metabolism, whereby intestinal microorganisms regulate the conversion of primary to secondary bile acids, while bile acids themselves shape microbial community structure and function [91,92]. Consequently, alcohol-induced disturbances in the gut microbiota may contribute to broader alterations in host metabolic homeostasis extending beyond the intestinal lumen [93]. The concomitant detection of changes in bile acids, lipid-related metabolites, and other microbiota-associated compounds further supports the view that alcohol-related dysbiosis affects multiple interconnected metabolic pathways rather than isolated microbial products.

One of the most distinctive findings identified in this review was the involvement of purine metabolism and adenosine signaling in alcohol-associated metabolic alterations. Using untargeted metabolomics, Wang et al. [75] reported significant changes in purine metabolism in individuals with long-term alcohol exposure and identified adenosine as one of the metabolites showing the strongest differences between alcohol-exposed participants and controls. Notably, purine metabolism emerged as a recurrent pathway in pathway enrichment analyses, and adenosine was the only metabolite consistently altered across the human gut metabolome and both gut and serum metabolomes in alcohol-exposed mice. In addition, the authors identified alcohol-associated changes in microbial genes involved in adenosine metabolism, including increased abundance of genes related to adenosine synthesis and reduced abundance of genes involved in adenosine degradation. Although the mechanistic relevance of these observations in humans remains to be established, these findings suggest that alcohol-induced alterations in gut microbial metabolism may extend beyond classical fermentation products and involve signaling molecules with potential systemic effects. Given the well-established role of purine metabolites in cellular signaling, immune regulation, and neurophysiological processes [94,95,96,97,98], these observations highlight purine metabolism as a potentially important but still insufficiently explored pathway in the context of alcohol-related gut dysbiosis (Figure 3).

The functional analyses available in a subset of studies further support the notion that alcohol-associated dysbiosis may have important metabolic consequences. In non-human primates, Phylogenetic Investigation of Communities by Reconstruction of Unobserved States (PICRUSt)-based predictions indicated enrichment of microbial pathways related to aromatic amino acid metabolism, branched-chain amino acid metabolism, purine biosynthesis, and tricarboxylic acid cycle activity in long-term drinkers [65]. Similarly, Blanton et al. [71] reported enrichment of predicted microbial pathways involved in fatty acid biosynthesis, while Blanton et al. [72] identified potential alterations in pathways related to formaldehyde assimilation and nucleotide metabolism following chronic alcohol exposure. In humans, Bjørkhaug et al. [76] observed an enrichment of predicted microbial functions associated with “bacterial invasion of epithelial cells” in a subset of alcohol overconsumers. Although these approaches provide indirect evidence and do not directly measure microbial activity, the predicted functional alterations were broadly consistent with the metabolomic changes observed across studies, including disturbances in amino acid metabolism, fatty acid metabolism, purine metabolism, and host–microbiota metabolic interactions. Together, these findings support the hypothesis that alcohol-related dysbiosis involves not only taxonomic shifts but also modifications in the metabolic functions performed by the gut microbiota, which may have direct consequences for host physiology.

Several findings identified in this review also support a potential involvement of the microbiota–gut–brain axis in alcohol-related pathophysiology. In addition to reporting marked alterations in microbiota-related metabolites, Leclercq et al. [39] observed that AD individuals with increased IP exhibited persistently elevated levels of depression, anxiety, and alcohol craving despite improvements in gut barrier function following abstinence. Extending these observations, Leclercq et al. [74] reported that increased IP was associated with lower sociability scores in the same cohort. More direct evidence linking microbiota-related metabolites with neuropsychiatric manifestations was provided by Leclercq et al. [79], who found that circulating concentrations of hippuric acid and pyrocatechol sulfate were negatively associated with depression, anxiety, and alcohol craving. Similarly, Kärkkäinen et al. [73] reported that reduced hippuric acid concentrations were associated with both alcohol consumption and depressive symptom severity. Importantly, Leclercq et al. [79] further demonstrated that several alcohol-sensitive metabolites, including hippuric acid, pyrocatechol sulfate, paraxanthine, theobromine, and 3-hydroxyvaleric acid, were detectable in postmortem cerebrospinal fluid and frontal cortex samples obtained from individuals with a history of heavy alcohol use. Although these observations do not establish causality, they suggest that alcohol-associated alterations in gut microbiota-related metabolites may extend beyond the intestinal environment and potentially influence biological pathways relevant to brain function and behavior. This interpretation is consistent with growing evidence supporting bidirectional communication between the gut microbiota and the CNS through metabolic, immune, endocrine, and neural pathways [18,91].

An additional finding emerging from both human and non-human primate studies is that alcohol-associated alterations in the gut microbiota and metabolome appear to be at least partially reversible following abstinence, although recovery is often incomplete. However, interpretation of these findings should consider the substantial variability in abstinence duration across the included studies. In non-human primates, recovery was evaluated only after short-term abstinence (3–5 days) [65,70], whereas the available human studies assessed abstinence periods ranging from approximately 3 weeks [39,74,79] to more than 6 weeks [78]. Consequently, differences in recovery patterns may reflect not only biological variability but also differences in follow-up duration. 

In AD individuals, abstinence was associated with partial restoration of microbial composition and with metabolomic changes that frequently occurred in the opposite direction to those observed during active alcohol use [39,79]. Similar findings were reported in non-human primates, where abstinence induced partial recovery of alcohol-associated microbial and metabolic alterations, particularly in pathways related to fatty acid and energy metabolism [70]. However, Piacentino et al. [65] found that long-term drinkers retained a distinct metabolomic profile during abstinence, suggesting that the effects of prolonged alcohol exposure may persist beyond short periods of withdrawal. Consistent with this interpretation, several alterations remained evident after abstinence in human studies, including persistent depletion of *F. prausnitzii* [39] and the marked microbiota and metabolomic alterations observed in abstinent individuals with AUD [78]. Interestingly, Forton et al. [87] found that circulating levels of the microbiota-derived tryptophan metabolites indole-3-carboxaldehyde and indole-3-acetic acid predicted relapse among individuals with AUD during early abstinence. Together with the recovery of indole-derived metabolites observed following alcohol withdrawal [79], these findings suggest that microbiota-related tryptophan metabolism may represent a dynamic biological pathway associated with both recovery and relapse risk. These observations further support the potential clinical utility of microbiota-derived metabolites as prognostic biomarkers during abstinence, although larger prospective studies are needed to validate their ability to predict relapse and treatment response. Collectively, these findings suggest that alcohol-associated alterations in the gut microbiota and metabolome are at least partially reversible, although the relatively short abstinence periods examined in the available studies indicate that complete recovery may require longer-term alcohol withdrawal (Figure 3).

Several methodological limitations inherent to the current body of evidence should also be considered. An important source of heterogeneity among the included studies was the considerable variation in analytical methodologies. Metabolomic profiling was performed using both targeted and untargeted approaches, including GC-MS, LC-MS/MS, ultra-high-performance liquid chromatography–quadrupole time-of-flight mass spectrometry (UHPLC-QTOF-MS), hydrophilic interaction liquid chromatography (HILIC)-based platforms, and fecal VOC profiling, each differing in metabolite coverage, sensitivity, analytical specificity, and quantitative capabilities. In particular, two studies characterized fecal VOCs using GC-MS-based approaches [39,77]. Although VOC profiling provides valuable information on microbial fermentation products and host–microbiota metabolic interactions, it captures a metabolite subset that differs substantially from conventional liquid chromatography-based metabolomics. Consequently, differences between analytical platforms may partly account for inconsistencies in the reported metabolomic signatures across studies. Likewise, microbiota characterization varied between studies, with most using 16S rRNA sequencing and one employing shotgun metagenomic sequencing [75], further limiting direct comparisons of taxonomic findings. 

Additional heterogeneity arose from the use of different biological matrices, including feces, plasma, serum, cerebrospinal fluid, and post-mortem brain tissue, each reflecting different aspects of host–microbiota metabolism. Furthermore, although alcohol exposure was consistently assessed using validated self-report instruments in human studies (e.g., Timeline Follow-Back, Alcohol Use Disorders Identification Test (AUDIT), quantity–frequency questionnaires, or DSM-based diagnostic criteria), these approaches remain susceptible to recall bias, social desirability bias, and underreporting of alcohol consumption. Such measurement error may have influenced the classification of drinking patterns and contributed to variability in the observed associations between alcohol exposure and metabolomic alterations. Collectively, these methodological differences should be considered when interpreting and comparing findings across studies and highlight the need for greater methodological standardization in future research.

An additional consideration for the translation of microbiota-derived metabolites into clinical biomarkers is the biological matrix used for their measurement. Fecal samples directly reflect gut microbial metabolic activity and therefore provide valuable mechanistic information, although sample collection, processing, and standardization remain challenging for routine clinical implementation. In contrast, plasma and serum metabolites are more readily accessible in clinical practice and may better reflect systemic metabolic alterations, facilitating longitudinal monitoring and biomarker validation [99]. Cerebrospinal fluid offers unique insight into gut–brain axis mechanisms and CNS exposure to microbiota-derived metabolites but is limited by the invasive nature of sample collection, restricting its applicability to specific clinical or research settings. Similarly, fecal VOC profiling represents a promising non-targeted approach for capturing microbial metabolic activity; however, methodological standardization, analytical reproducibility, and cost-effectiveness require further evaluation before widespread clinical adoption [77,100]. Consequently, future biomarker development should consider not only biological relevance but also sampling feasibility, analytical cost, and clinical scalability when selecting candidate metabolite signatures.

Taken together, the studies included in this review support the concept that alcohol consumption is associated with alterations in gut microbiota composition and, more consistently, with disruptions in microbiota-related metabolic pathways. Across human and non-human primate studies, recurrent changes were identified in SCFAs, tryptophan-derived metabolites, phenolic compounds, bile acids, and purine-related metabolites, suggesting that alcohol exposure may affect multiple interconnected metabolic processes at the host–microbiota interface. Importantly, several of these metabolites were associated with IP, psychological symptoms, alcohol craving, or were detected in CNS compartments, highlighting their potential relevance beyond the gastrointestinal tract. While the available evidence does not allow causal inferences, it supports the hypothesis that microbiota-derived metabolites may represent key mediators linking alcohol exposure to intestinal, metabolic, and neurobehavioral alterations. Further longitudinal and multi-omics studies integrating microbiome, metabolome, and clinical phenotyping data will be necessary to clarify the temporal dynamics, mechanistic significance, and potential clinical utility of these pathways in alcohol-related disorders.

## 5. Conclusions

### 5.1. General Conclusions

This systematic review demonstrates that alcohol consumption is consistently associated with alterations in the functional activity of the gut microbiota in both humans and non-human primates. Although changes in microbial diversity and taxonomic composition were heterogeneous across studies, microbiota-derived metabolite profiles showed greater consistency, suggesting that functional metabolic alterations may represent more robust biomarkers of alcohol exposure than taxonomic composition alone.

Among the metabolites identified, reductions in SCFAs, alterations in tryptophan-derived metabolites, and changes in hippuric acid emerged as the most consistent findings across both human and non-human primate studies, whereas disturbances in bile acid and purine metabolism were also recurrently reported. 

Collectively, these alterations support the hypothesis that alcohol disrupts the functional output of the gut microbiota, with downstream effects on intestinal barrier integrity, immune regulation, liver function, and gut–brain communication. Supporting this concept, several microbiota-derived metabolites were associated with intestinal permeability, psychological symptoms, and alcohol craving or were detected in CNS compartments, underscoring their potential value as functional biomarkers of alcohol-related pathophysiology beyond the gastrointestinal tract.

Evidence from abstinence studies further indicates that alcohol-associated microbial and metabolomic alterations are only partially reversible during the early stages of alcohol withdrawal. The persistence of several microbiota and metabolomic changes after abstinence, together with emerging evidence that microbiota-derived tryptophan metabolites may predict relapse risk, highlights their potential value as prognostic biomarkers in AUD.

Overall, despite the predominantly associative nature of the available evidence, the consistent metabolomic signatures identified across independent studies provide a strong rationale for further longitudinal and interventional research aimed at validating microbiota-derived metabolites as clinically relevant biomarkers and therapeutic targets in alcohol-related disorders.

### 5.2. Study Limitations

Several limitations of the available evidence should be considered when interpreting the findings of this review. First, the number of eligible studies was relatively small, partly due to the exclusion of studies focused exclusively on severe alcohol-related comorbidities, particularly alcohol-related liver disease. Although this reduced the available evidence base, it also represents a methodological strength, as it allowed a more specific evaluation of alcohol-associated microbiota and metabolomic alterations while minimizing the confounding influence of advanced disease processes. Second, substantial heterogeneity existed across study designs, populations, alcohol exposure patterns, biological matrices, and analytical methodologies. The included studies assessed metabolites in fecal samples, plasma, serum, cerebrospinal fluid, and brain tissue using a variety of targeted and untargeted metabolomic approaches, which complicates direct comparisons across studies. Moreover, although integrating evidence from non-human primates and humans provides valuable translational insights, direct comparisons between these models should be interpreted with caution. Non-human primate studies were conducted under tightly controlled experimental conditions with standardized alcohol exposure, whereas human studies were inherently more heterogeneous with respect to drinking patterns, lifestyle, diet, comorbidities, and other environmental factors. Consequently, cross-species comparisons should be regarded as complementary rather than directly equivalent. Third, most human studies were cross-sectional, limiting the ability to determine temporal relationships or infer causality between alcohol exposure, microbial alterations, and metabolomic changes. Fourth, several studies included relatively small sample sizes, potentially reducing statistical power and limiting generalizability. Collectively, the limited number of eligible studies and the substantial methodological heterogeneity precluded the performance of a quantitative meta-analysis.

In addition, important factors known to influence the gut microbiota and metabolome, including diet, smoking status, medication use, and comorbid conditions, were not consistently controlled across studies. Finally, only a subset of investigations simultaneously assessed gut microbiota composition and metabolite profiles, limiting the ability to directly link specific microbial taxa with the observed metabolic alterations. Although the overall methodological quality of the included studies was moderate to high, limitations related to study comparability in human cohorts and incomplete reporting of randomization and blinding procedures in non-human primate studies should be considered when interpreting the available evidence.

### 5.3. Future Research Directions

Future research should therefore be prioritized across three complementary translational tiers—basic non-human primate multi-omics studies, large-scale human clinical validation, and therapeutic target development—to clarify causal relationships and establish the translational relevance of these metabolites in alcohol-related disorders.

The non-human primate studies included in this review provide a controlled model for isolating the effects of alcohol exposure from confounding factors inherent to human cohorts (e.g., diet, medication use, comorbidities), but most available data derive from cross-sectional or short-term designs. Future work should prioritize longitudinal multi-omics studies combining 16S/shotgun metagenomics, targeted and untargeted metabolomics, and, where feasible, transcriptomic or host proteomic profiling, across sequential phases of alcohol exposure, dependence, and abstinence in the same animals. Beyond static metabolite profiling, future studies should also consider incorporating stable isotope-resolved metabolomics (SIRM), using isotopically labeled dietary substrates to trace microbial metabolic fluxes contributing to fecal and plasma pools [101,102]. This approach would allow the alterations identified here to be attributed more directly to microbial activity rather than inferred from static concentrations. Such an approach has a well-established safety profile and could potentially be incorporated into existing voluntary self-administration paradigms without additional invasive procedures, although its application to gut microbiome flux has so far been explored primarily in rodent models [101]. Particular attention should be paid to extending abstinence monitoring beyond the short windows evaluated in the studies reviewed here, to determine whether the partial recovery patterns observed reflect a genuine ceiling of reversibility or an artifact of insufficient follow-up.

Most human evidence reviewed here derives from cross-sectional studies with heterogeneous biological matrices and inconsistent control of confounding factors precluding causal inference. Large, prospective population-based cohorts with linked microbiome and metabolome data already provide the infrastructure needed to overcome these limitations. Among the available cohorts, the Finnish FINRISK 2002 study has generated shotgun metagenomic and plasma metabolomic profiles from over 7000 participants with up to 18 years of register-based follow-up and has recently been used to establish prospective associations between alcohol intake, gut microbiome composition, and clinical outcomes [103]. Applying this type of design specifically to the recurrent metabolite panel identified in the present review, including SCFAs, tryptophan-derived metabolites, hippuric acid, bile acids, and purine-related metabolites, using mediation analyses to link alcohol exposure, microbiota-derived metabolites, and incident alcohol-related outcomes, would allow the field to move beyond cross-sectional associations toward temporally resolved, population-level evidence. Furthermore, replicating these findings across independent cohorts and diverse ancestral backgrounds would help address the limited ethnic and geographic diversity of the studies included in this review. 

Future studies should also establish standardized targeted metabolomic panels in clinically accessible biological matrices, particularly plasma, while defining the complementary role of fecal and cerebrospinal fluid biomarkers according to their biological and clinical applications. Harmonizing analytical platforms and quantification strategies would facilitate direct comparisons across cohorts and accelerate validation of candidate biomarkers. Another important priority will be to strengthen evidence that the metabolites identified are mechanistically linked to gut microbial activity rather than reflecting host metabolism alone. Future studies combining metagenomic functional profiling with isotope tracing, microbial gene abundance, or ex vivo fermentation experiments could substantially improve causal attribution. 

Finally, once robust mechanistic links between alcohol exposure, microbial metabolism, and host physiology have been established, future interventional studies should evaluate whether modulation of the microbial metabolic pathways identified in this review, through dietary interventions, prebiotics, probiotics, postbiotics, or targeted microbial enzyme modulation, can attenuate alcohol-induced tissue injury and improve clinical outcomes. Initial clinical evidence already supports the feasibility of this approach. A randomized controlled trial in patients with alcohol use disorder demonstrated that prebiotic inulin supplementation during alcohol withdrawal modified both fecal and plasma microbiota-derived metabolomic profiles, with several of these metabolite changes correlating with markers of liver function and metabolic status [104]. More recently, a randomized, double-blind, placebo-controlled trial showed that supplementation with *Weizmannia coagulans* BC99 increased butyrate-related metabolites while improving gut barrier integrity, enhancing alcohol metabolism, and reducing oxidative stress and systemic inflammation in chronic alcohol consumers [105]. As additional interventional evidence emerges, integrating metabolomic profiling with functional and clinical outcomes will be essential to move beyond associative evidence, establish causal relationships, and identify microbiome-informed therapeutic strategies for alcohol-related disorders.

Together, these complementary translational approaches will help bridge the gap between descriptive associations and mechanistic understanding, ultimately facilitating the validation of robust biomarkers and mechanistically informed microbiome-targeted therapeutic strategies for alcohol-related disorders.

## Figures and Tables

**Table 2:** Quality of human studies: Newcastle–Ottawa Scale.

Study	Selection	Comparability	Outcome	Overall	Quality
Couch et al. (2015) [77]	***	*	***	7/9	High
Leclercq et al. (2014) [39]	****	**	***	9/9	High
Wang et al. (2026) [75]	****	*	**	7/9	High
Bjorkhaug et al. (2019) [76]	***	*	**	6/9	Moderate
Kärkkäinen et al. (2024) [73]	**	*	**	5/9	Moderate
Piacentino et al. (2024) [78]	****	**	***	9/9	High
Leclercq et al. (2024) [79]	****	**	***	9/9	High
Leclercq et al. (2020) [74]	****	**	***	9/9	High

Note: Each asterisk (*) represents one point on the Newcastle–Ottawa Scale. Maximum scores: Selection = 4, Comparability = 2, Outcome = 3 (total max = 9). Quality: High = 7–9, Moderate = 4–6, Low = 0–3.

## Data Availability

No new data were created or analyzed in this study. Data sharing is not applicable to this article.

## Abbreviations

The following abbreviations are used in this manuscript:

AD	Alcohol Dependence
AhR	Aryl Hydrocarbon Receptor
ASV	Amplicon Sequence Variant
AUD	Alcohol Use Disorder
BAL	Blood Alcohol Level
BBB	Blood–Brain Barrier
BEC	Blood Ethanol Concentration
BHB	β-Hydroxybutyrate
CNS	Central Nervous System
CSF	Cerebrospinal Fluid
DSM	Diagnostic and Statistical Manual of Mental Disorders
FDR	False Discovery Rate
GC–MS	Gas Chromatography–Mass Spectrometry
HILIC	Hydrophilic Interaction Liquid Chromatography
IL	Interleukin
IP	Intestinal Permeability
LC-MS/MS	Liquid Chromatography–Tandem Mass Spectrometry
LEfSe	Linear Discriminant Analysis Effect Size
LPS	Lipopolysaccharide
NEFA	Non-Esterified Fatty Acid
NHP	Non-Human Primate
NOS	Newcastle–Ottawa Scale
PCA	Principal Component Analysis
PCoA	Principal Coordinates Analysis
PICRUSt	Phylogenetic Investigation of Communities by Reconstruction of Unobserved States
PLS-DA	Partial Least Squares Discriminant Analysis
PRISMA	Preferred Reporting Items for Systematic Reviews and Meta-Analyses
qPCR	Quantitative Polymerase Chain Reaction
ROS	Reactive Oxygen Species
SCFA	Short-Chain Fatty Acid
SYRCLE	Systematic Review Centre for Laboratory Animal Experimentation
TLFB	Timeline Follow-Back
TNFα	Tumor necrosis factor alpha
VOC	Volatile Organic Compound
